# Effort-reward imbalance and emotional exhaustion among border-region physical education teachers: the roles of meaningful work and social support

**DOI:** 10.3389/fpsyg.2026.1883202

**Published:** 2026-10-05

**Authors:** Yintu Bao, Zhaohua Sun, Hongbin Yue, Boya Dong, Changliang Yan

**Affiliations:** 1 Physical Education Teaching Department, Dalian Minzu University, Dalian, China; 2 Liaoning Police Academy, Dalian, China; 3 Department of Physical Education, Northeastern University, Shenyang, China

**Keywords:** effort–reward imbalance, emotional exhaustion, meaningful work, physical education teachers, social support

## Abstract

**Introduction:**

Emotional exhaustion has become a prevalent psychological concern among teachers working in high-demand educational environments. Drawing upon effort–reward imbalance (ERI) theory and conservation of resources theory, this study examined the relationship between ERI and emotional exhaustion among teachers working in border regions of China, with meaningful work as a mediator and social support as a moderator.

**Methods:**

A cross-sectional survey was conducted among 483 primary and secondary school physical education teachers. Data were analyzed using PROCESS Models 4 and 14 with 5,000 bootstrap resamples.

**Results:**

The results indicated that ERI was positively associated with emotional exhaustion. Meaningful work accounted for a significant indirect association between ERI and emotional exhaustion. In addition, social support moderated the association between meaningful work and emotional exhaustion, such that the positive association was weaker at higher levels of social support. The conditional indirect association between ERI and emotional exhaustion through meaningful work was also smaller at higher levels of social support.

**Discussion:**

These findings highlight the complex psychological role of meaningful work under chronically demanding work conditions and emphasize the importance of supportive school environments for maintaining teachers’ psychological well-being.

## 1 Introduction

Emotional exhaustion, as the core component of burnout, reflects the depletion of individuals’ emotional and energetic resources under prolonged job stress (Maslach and Leiter, 2016). In educational settings, emotional exhaustion has been consistently associated with reduced teaching effectiveness, impaired psychological well-being, and increased turnover intentions (Madigan and Kim, 2021). In sport-related professions, emotional exhaustion has also been identified as the most central and sensitive indicator of burnout because it directly captures the energy depletion caused by overwhelming demands (Yan et al., 2023). Therefore, focusing on emotional exhaustion provides a theoretically grounded and contextually appropriate approach for understanding stress-related outcomes among physical education (PE) teachers.

Compared with teachers in other subject areas, PE teachers often face more complex and physically demanding work conditions. In addition to regular teaching responsibilities, they are frequently required to undertake extracurricular training, competition organization, and student safety supervision, resulting in elevated workload and role complexity (Alsalhe et al., 2021). These multiple responsibilities may increase exposure to sustained job demands and place PE teachers at greater risk of emotional exhaustion.

Importantly, burnout is shaped not only by individual characteristics but also by contextual conditions (Yan et al., 2023). Prior research has shown that high job demands, insufficient resources, and limited institutional support are associated with burnout. Rural education systems may experience teacher shortages and institutional inequalities (UNESCO, 2021). However, the present study did not compare border-region teachers with teachers in other locations and therefore does not assume that these conditions are unique to border regions.

The participants were recruited from schools located in border regions of China. This geographic feature is treated as a characteristic of the sampling frame rather than as a tested component of the theoretical model. The study does not measure geographic dispersion, ethnic composition, or cross-cultural contact and therefore does not infer that these features explain the observed associations.

From a theoretical perspective, the effort–reward Imbalance (ERI) model provides an important framework for understanding occupational stress (Siegrist, 1996). According to the ERI model, stress emerges when high levels of effort are not matched by adequate rewards, thereby violating the principle of reciprocity and generating sustained psychological strain. Extensive empirical evidence has demonstrated that ERI is positively associated with burnout, particularly emotional exhaustion (van Vegchel et al., 2005).

Meaningful work is generally regarded as a positive psychological resource that promotes motivation, engagement, and resilience (Steger et al., 2012). However, recent research suggests that meaningful work may also involve potential psychological costs. Individuals who strongly value the meaning of their work may continue investing substantial effort even under unfavorable working conditions (Bunderson and Thompson, 2009; Bailey et al., 2017). Prior studies have further indicated that strong work meaning is associated with persistence, compulsive work tendencies, and excessive personal investment (Schaufeli et al., 2002; Van Beek et al., 2012). Although such persistence may sustain work engagement, prolonged resource investment without adequate recovery may accelerate emotional and energetic depletion (Halbesleben and Buckley, 2004).

This pattern may be particularly salient in highly constrained work contexts. When opportunities for external adjustment are limited, individuals who perceive their work as meaningful may be more likely to maintain effort investment despite ongoing imbalance and stress. Over time, such meaning-driven persistence may contribute to emotional exhaustion through continuous resource depletion.

In addition to individual motivational processes, contextual resources may be associated with variation in occupational-stress outcomes. Social support, defined as emotional, informational, and instrumental assistance received from others, is widely recognized as a resource relevant to coping with work-related stress (Cohen and Wills, 1985). Within the Job Demands–Resources framework, higher social support is theorized to coincide with weaker associations between job demands and resource loss (Bakker and Demerouti, 2007). In the present cross-sectional study, this proposition is evaluated as a conditional association and not as a causal buffering effect.

Accordingly, the present study aims to examine the relationship between effort–reward imbalance and emotional exhaustion among PE teachers in Chinese border regions. In particular, this study investigates the mediating role of meaningful work and the moderating role of social support within this process.

## 2 Hypotheses development

### 2.1 Effort–reward imbalance and emotional exhaustion

According to the effort–reward imbalance (ERI) model, occupational stress emerges when individuals invest substantial effort without receiving adequate rewards in return (Siegrist, 1996). Such imbalance violates the norm of reciprocity and produces sustained psychological strain, which may gradually deplete emotional and energetic resources. Prior research has consistently demonstrated that ERI is positively associated with burnout, particularly emotional exhaustion (van Vegchel et al., 2005).

For PE teachers, effort–reward imbalance may be relevant because their work can involve physical demands and multiple professional roles. The present study examines this occupational-stress association in a sample recruited from border-region schools; it does not test whether the association differs from that observed in non-border schools.

Therefore, the following hypothesis is proposed:

**H1:** Effort–reward imbalance is positively associated with emotional exhaustion.

### 2.2 Effort–reward imbalance and meaningful work

Meaningful work refers to the perception that one’s work is personally significant and valuable (Steger et al., 2012). Traditionally, meaningful work has been viewed as a beneficial psychological resource associated with positive work attitudes and well-being. However, recent studies suggest that individuals who strongly value the meaning of their work may also demonstrate stronger persistence and continued effort investment under adverse conditions (Bunderson and Thompson, 2009; Bailey et al., 2017).

In demanding work environments, employees may continue investing effort despite unfavorable conditions because they perceive their work as important and worthwhile. In educational contexts, teachers often attach moral and social value to their professional roles, which may coexist with continued commitment even when rewards are perceived as insufficient. The proposed positive association between effort–reward imbalance and meaningful work is therefore treated as a theory-guided possibility rather than a demonstrated temporal process.

The present study examines this proposed association among PE teachers recruited from schools in China’s border regions. Border-region recruitment defines the sampling setting; it is not treated as evidence that the setting is uniquely constrained or that contextual characteristics caused the measured associations.

Accordingly, the following hypothesis is proposed:

**H2:** Effort–reward imbalance is positively associated with meaningful work.

### 2.3 Meaningful work and emotional exhaustion

Although meaningful work is commonly regarded as beneficial, emerging evidence suggests that it may also involve psychological costs under certain conditions. Individuals who perceive their work as highly meaningful are often more willing to persist, tolerate hardship, and invest substantial personal resources in their work (Bunderson and Thompson, 2009).

Previous studies have shown that strong work meaning is associated with compulsive work tendencies, excessive effort investment, and difficulty disengaging from work responsibilities (Schaufeli et al., 2002; Van Beek et al., 2012). From the perspective of Conservation of Resources (COR) theory, continuous resource investment without adequate recovery may accelerate emotional depletion and contribute to burnout (Hobfoll, 1989).

In high-demand and resource-constrained contexts, individuals who strongly value the meaning of their work may continue investing effort despite ongoing stress and imbalance. Although such persistence may sustain engagement in the short term, prolonged meaning-driven investment may gradually increase emotional exhaustion through continuous resource depletion.

Therefore, the following hypothesis is proposed:

**H3:** Meaningful work is positively associated with emotional exhaustion.

### 2.4 The mediating role of meaningful work

Integrating the above arguments, meaningful work may statistically account for part of the association between effort–reward imbalance and emotional exhaustion. Under the proposed ordering, perceived imbalance may coexist with continued meaning-based investment, and such sustained investment may in turn be associated with greater emotional burden. This specification is a theory-guided indirect-association model rather than evidence that meaningful work temporally transmits a causal effect.

Alternative orderings are equally compatible with the cross-sectional data. Teachers who already experience their work as highly meaningful may perceive their effort as more justified, evaluate available rewards differently, or report effort–reward imbalance differently. Meaningful work could also operate as a moderator, such that the association between imbalance and exhaustion varies across levels of perceived meaning, rather than as an intervening variable. Because all variables were measured concurrently, the present study cannot distinguish mediation, moderation, reverse-direction, or reciprocal processes.

Accordingly, the following hypothesis is proposed:

**H4:** Meaningful work mediates the relationship between effort–reward imbalance and emotional exhaustion.

### 2.5 The moderating role of social support

Social support refers to emotional, informational, and instrumental assistance provided by others (Cohen and Wills, 1985). Within the Job Demands–Resources (JD-R) framework, social support is considered an important job resource that can buffer the negative effects of job demands and reduce resource depletion (Bakker and Demerouti, 2007).

Importantly, social support may be associated with variation in the relationship between meaningful work and emotional exhaustion. At higher levels of social support, individuals may report sustained work engagement alongside lower emotional costs, whereas insufficient support may coincide with greater reliance on internal motivational resources and higher emotional depletion. These propositions concern conditional associations and do not establish a causal buffering process.

Therefore, social support may weaken the positive relationship between meaningful work and emotional exhaustion.

Accordingly, the following hypothesis is proposed:

**H5:** Social support moderates the relationship between meaningful work and emotional exhaustion, such that the positive relationship is weaker when social support is high.

The conceptual model is presented in Figure 1.

**FIGURE 1:**
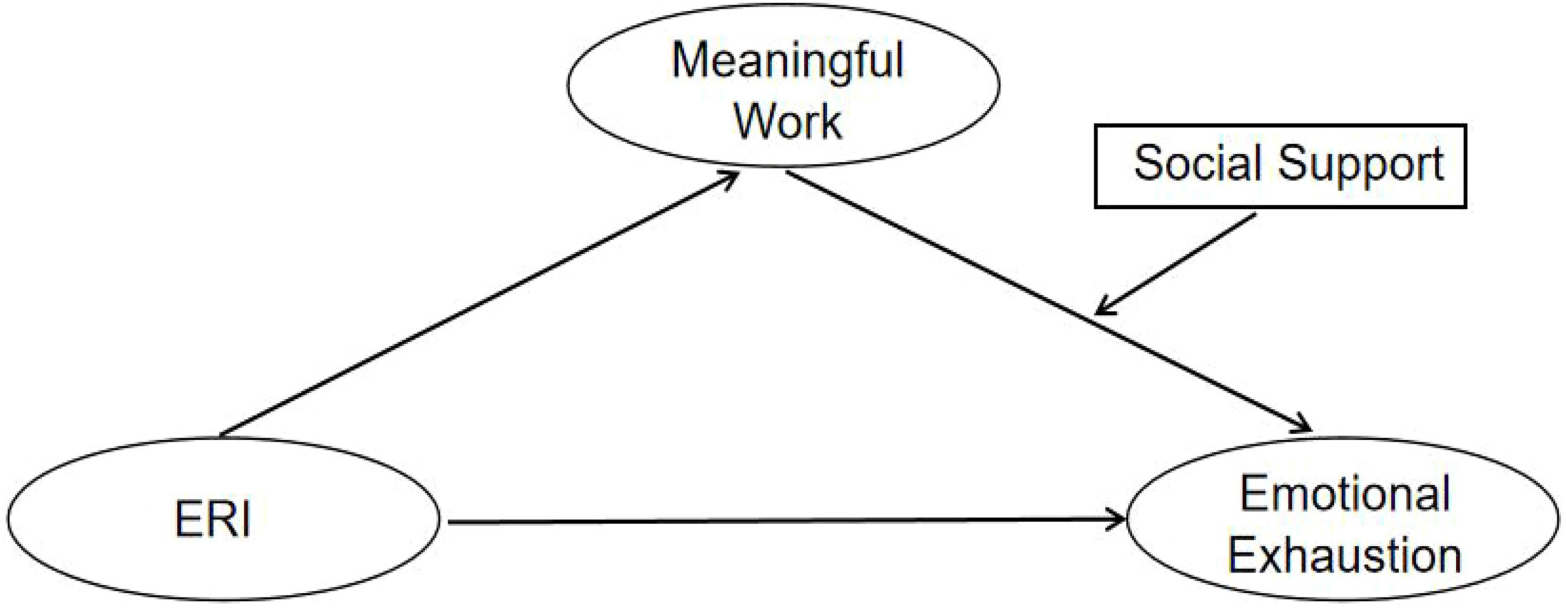
Conceptual model of the present study.

## 3 Materials and methods

### 3.1 Participants and procedure

This study employed a cross-sectional questionnaire design to examine the relationships among effort–reward imbalance (ERI), meaningful work, social support, and emotional exhaustion among physical education (PE) teachers in the border regions of China. A non-probability convenience sampling strategy was used to recruit PE teachers from primary and secondary schools in the border areas of Liaoning, Jilin, and Heilongjiang Provinces.

Data were collected using a combination of online and paper-based questionnaires. Before completing the questionnaire, all participants were informed of the purpose of the study, the voluntary nature of participation, the confidentiality and anonymity of their responses, and their right to withdraw at any time without penalty. Informed consent was obtained from all participants prior to participation.

The study involved human participants and received ethical approval from the Bioethics Committee of Northeastern University (Approval No. EC-2024B034) before data collection. All participants provided informed consent.

A total of 500 questionnaires were distributed. Before analysis, the returned questionnaires were screened for data quality. Questionnaires were excluded if they showed invariant or clearly patterned responding across the instrument, contained missing responses that prevented computation of a scale score, or had an implausibly short completion time relative to the questionnaire length. Seventeen questionnaires were excluded, leaving 483 valid questionnaires and yielding a valid response rate of 96.6%. Of the valid questionnaires, 178 were collected online (36.9%) and 305 were collected offline in paper-and-pencil format (63.1%). The retained questionnaires contained no missing values requiring statistical imputation.

As presented in Table 1, 297 participants were male (61.5%) and 186 were female (38.5%). Regarding teaching experience, 25.1% had 6–10 years of experience and 23.0% had 11–15 years. Most participants held a bachelor’s degree (69.8%), whereas 14.5% held a master’s degree or above. Regarding school location, 39.8% worked in township schools and 25.5% worked in rural or remote schools.

**TABLE 1:** Demographic characteristics of the sample (*N* = 483).

Variable	Category	*n*	%
Gender	Male	297	61.5
	Female	186	38.5
Teaching experience	≤5 years	92	19.0
	6–10 years	121	25.1
	11–15 years	111	23.0
	16–20 years	84	17.4
	≥21 years	75	15.5
Age	≤30 years	98	20.3
	31–40 years	176	36.4
	41–50 years	139	28.8
	≥51 years	70	14.5
Education level	College degree or below	76	15.7
	Bachelor’s degree	337	69.8
	Master’s degree or above	70	14.5
School location	Urban or county-area school	168	34.8
	Township school	192	39.8
	Rural or remote school	123	25.5

PE, physical education. Percentages may not total 100 because of rounding.

### 3.2 Measures

All measures were administered in Chinese. Following established cross-cultural adaptation procedures, the original English items were independently translated into Chinese by two bilingual researchers. A separate bilingual researcher, who had not seen the original items, then back-translated the Chinese version into English. Discrepancies were resolved through discussion. Two experts in occupational health psychology and one experienced PE teacher subsequently reviewed the items for semantic equivalence, content relevance, and contextual appropriateness. The resulting questionnaire was pilot-tested with a small group of PE teachers to assess clarity and comprehensibility, and minor wording adjustments were made before formal data collection.

#### 3.2.1 Effort–reward imbalance

Effort–reward imbalance (ERI) was assessed using the short ERI Questionnaire developed within the model proposed by Siegrist (1996) and further documented by Siegrist et al. (2014). The instrument comprised three Effort items and seven Reward items. Effort and Reward were scored as two constituent subscales and modeled as separate latent factors in the confirmatory factor analysis. Representative items included “I have constant time pressure due to a heavy workload” and “I receive the respect I deserve from my supervisors or relevant others.” For the regression and PROCESS analyses, the conventional ERI ratio was used to represent non-reciprocity between the two components; separate Effort and Reward predictor models were additionally estimated as a sensitivity analysis.

All items were rated on a four-point Likert scale ranging from 1 to 4. Following the standard ERI procedure, the ratio was calculated from summed scores using the formula:


ERI=Effort/(Reward×3/7)

Because the correction factor adjusts for the unequal numbers of Effort and Reward items, E_sum/(R_sum × 3/7) is algebraically equivalent to mean Effort/mean Reward in the present data. Higher ERI values indicated greater imbalance, and values above 1 indicated that reported effort exceeded reported reward on the common response metric. The ratio was retained for the primary analyses because ERI theory conceptualizes strain as a mismatch between the two components. Cronbach’s alpha coefficients were 0.796 for Effort and 0.928 for Reward; McDonald’s omega coefficients were 0.801 and 0.930, respectively.

#### 3.2.2 Meaningful work

Meaningful work was measured using five positively worded items adapted from the five-item Work Meaningfulness measure used by Bunderson and Thompson (2009). The measure assesses the extent to which individuals regard their work as important, personally meaningful, and capable of contributing to a broader purpose. Representative items included “I believe my work is meaningful” and “The work I do serves a greater purpose.” In the present study, the items were translated into Chinese, reviewed for semantic and contextual suitability for physical education teachers, and pilot-tested for clarity; no item was deleted from the five-item set.

Items were rated on a five-point Likert scale, with higher mean scores indicating stronger perceptions of meaningful work. The five items used in the present study were positively worded; therefore, no reverse scoring was required. In the current sample, the scale demonstrated good reliability (Cronbach’s α = 0.875; McDonald’s ω = 0.876).

#### 3.2.3 Emotional exhaustion

Emotional exhaustion was assessed using the five-item Exhaustion subscale of the Maslach Burnout Inventory–General Survey (MBI-GS; Schaufeli et al., 1996). Exhaustion is a constituent dimension of burnout rather than an independent burnout scale. The five items assess work-related emotional depletion, end-of-day exhaustion, fatigue when facing another workday, strain arising from sustained work demands, and an overall sense of being exhausted by one’s work. The item content was translated into Chinese and reviewed for contextual suitability, and the response format used in this study was a five-point Likert scale. Higher mean scores indicated greater emotional exhaustion. The subscale demonstrated high reliability in the present sample (Cronbach’s α = 0.898; McDonald’s ω = 0.901).

#### 3.2.4 Social support

Social support was assessed using six items adapted from the workplace social-support questions described by House and Wells (1978) and subsequently discussed by House (1981). The original approach assesses whether relevant people can be relied upon, are willing to listen to work-related problems, and provide practical or emotional assistance. For the present study, references to support providers were expressed in general terms relevant to physical education teachers, and minor wording adjustments were made following bilingual review and pilot testing. Representative items included “When I talk about my troubles, others are willing to listen” and “Others make me feel that I am important and valued.” All six items were retained and rated on a five-point Likert scale, with higher mean scores indicating greater perceived social support. The scale demonstrated excellent reliability in the present sample (Cronbach’s α = 0.928; McDonald’s ω = 0.929).

### 3.3 Data analysis

All statistical analyses were conducted using IBM SPSS 27.0, AMOS 24.0, and PROCESS Macro 4.2 developed by Andrew F. Hayes. Preliminary screening included inspection of univariate distributions and influential multivariate observations. Skewness and kurtosis were examined for the observed variables, and Mahalanobis distance was used as a diagnostic for potentially influential multivariate cases. Potentially influential cases were examined rather than automatically deleted. Descriptive statistics, reliability analyses, and Pearson correlation analyses were then performed to examine the distributions and interrelationships among the study variables.

Second, confirmatory factor analysis (CFA) was conducted using AMOS 24.0 to evaluate the construct validity of the measurement model. Model fit was assessed using multiple fit indices, including the comparative fit index (CFI), Tucker–Lewis index (TLI), root mean square error of approximation (RMSEA), and standardized root mean square residual (SRMR). Following commonly accepted criteria, CFI and TLI values above 0.90 and RMSEA and SRMR values below 0.08 were considered indicative of acceptable model fit (Hu and Bentler, 1999).

Reliability was evaluated using Cronbach’s alpha coefficients and McDonald’s omega coefficients. Convergent validity was assessed through composite reliability (CR) and average variance extracted (AVE).

To estimate the indirect association involving meaningful work, PROCESS Model 4 developed by Andrew F. Hayes was employed using 5,000 bootstrap resamples and 95% confidence intervals. PROCESS Model 14 was then applied to estimate the conditional indirect association, with social support specified as a moderator of the relationship between meaningful work and emotional exhaustion. Prior to constructing the interaction term, the continuous variables involved in the interaction were mean-centered to facilitate interpretation of the interaction term and the associated lower-order coefficients.

Gender, age, teaching experience, education level, and school location were included as control variables because prior research has suggested that demographic and occupational characteristics may influence teachers’ emotional exhaustion. For categorical variables with more than two groups, dummy coding procedures were applied before inclusion in the regression models.

Conditional indirect effects at different levels of social support were further examined using bootstrap confidence intervals. An indirect effect was considered statistically significant when the 95% confidence interval did not include zero.

Because all variables were collected concurrently using self-report questionnaires, common method variance was addressed through procedural safeguards and assessed *post hoc* using Harman’s single-factor test. Procedural safeguards included anonymous participation, assurances that there were no correct or incorrect responses, and separation of the focal constructs into distinct questionnaire sections. The statistical result of the post-hoc assessment is reported in the Results section.

## 4 Results

### 4.1 Measurement model

Confirmatory factor analysis (CFA) was conducted using AMOS 24.0 to examine the construct validity of the measurement model. In this analysis, Effort and Reward were modeled as separate constituent latent factors, together with Meaningful Work, Emotional Exhaustion, and Social Support; the calculated ERI ratio was used only in the subsequent regression and PROCESS analyses. The hypothesized five-factor model demonstrated satisfactory fit to the data, χ^2^(289) = 618.274, *p* < 0.001, χ^2^/df = 2.139, comparative fit index (CFI) = 0.960, Tucker–Lewis index (TLI) = 0.955, goodness-of-fit index (GFI) = 0.914, root mean square error of approximation (RMSEA) = 0.049, and standardized root mean square residual (SRMR) = 0.041.

According to commonly accepted criteria (Hu and Bentler, 1999), the results indicated that the measurement model demonstrated good construct validity and acceptable overall model fit.

Composite reliability (CR) and average variance extracted (AVE) were further calculated to assess convergent validity and construct reliability. As shown in Table 2, all CR values exceeded the recommended threshold of 0.70, and all AVE values were above 0.50, indicating satisfactory convergent validity and internal consistency reliability.

**TABLE 2:** Reliability and convergent validity.

Construct	α	ω	CR	AVE
Effort	0.796	0.801	0.801	0.575
Reward	0.928	0.930	0.930	0.657
Meaningful work	0.875	0.876	0.877	0.591
Emotional exhaustion	0.898	0.901	0.901	0.646
Social support	0.928	0.929	0.929	0.685

### 4.2 Common method variance assessment

An unrotated principal-component analysis of all 26 measurement items showed that the first component accounted for 30.110% of the total variance, below the commonly used 50% reference value. Thus, no single component dominated the covariance among the items. This result provides limited evidence that common method variance was not the predominant source of the observed associations. Nevertheless, Harman’s single-factor test is only a coarse post-hoc diagnostic and cannot rule out common method bias; accordingly, the findings remain conditional associations rather than causal effects.

### 4.3 Descriptive statistics and correlations

Means, standard deviations, and correlations among the study variables are presented in Table 3. Effort–reward imbalance (ERI) was positively correlated with meaningful work (*r* = 0.441, *p* < 0.001) and emotional exhaustion (*r* = 0.415, *p* < 0.001). Meaningful work was also positively associated with emotional exhaustion (*r* = 0.390, *p* < 0.001). Social support was not significantly correlated with ERI, meaningful work, or emotional exhaustion.

**TABLE 3:** Means, standard deviations, and correlations among variables.

Variable	M	SD	1	2	3	4
ERI	1.44	0.74	–	–	–	–
Meaningful work	3.68	0.98	0.441***			
Emotional exhaustion	3.57	0.94	0.415***	0.390***		
Social support	2.61	1.02	−0.082	0.072	0.008	

*N* = 483. ERI, effort–reward imbalance. ***p < 0.001.

### 4.4 Testing the mediating effect of meaningful work

PROCESS Model 4 developed by Hayes (2022) was used to estimate the indirect association involving meaningful work. As shown in Table 4, ERI was positively associated with meaningful work (*b* = 0.5827, SE = 0.0525, *t* = 11.1046, *p* < 0.001), and meaningful work was positively associated with emotional exhaustion after accounting for ERI and the covariates (*b* = 0.2287, SE = 0.0394, *t* = 5.8017, *p* < 0.001).

**TABLE 4:** Mediation analysis results.

Path	*b*	SE	t	*P*	LLCI	ULCI
ERI → Meaningful work	0.5827	0.0525	11.1046	<0.001	0.4796	0.6859
Meaningful work → Emotional exhaustion	0.2287	0.0394	5.8017	<0.001	0.1513	0.3062
ERI → Emotional exhaustion (direct effect)	0.3563	0.0539	6.6075	<0.001	0.2503	0.4622

The conditional direct association between ERI and emotional exhaustion remained statistically significant (*b* = 0.3563, SE = 0.0539, *t* = 6.6075, *p* < 0.001). Bootstrap analyses based on 5,000 resamples indicated a statistically significant indirect association between ERI and emotional exhaustion through meaningful work because the 95% confidence interval did not include zero. These estimates represent model-based associations and do not establish temporal ordering or causation.

As a sensitivity analysis, Effort and Reward were entered as separate predictors using the demographic variables available in the archived analysis file (gender, age, and teaching experience) as covariates. Both components were independently associated with meaningful work (Effort: *b* = 0.4455, SE = 0.0522, *t* = 8.54, *p* < 0.001; Reward: *b* = −0.5650, SE = 0.0546, *t* = −10.35, *p* < 0.001). In the emotional-exhaustion model that also included meaningful work, Effort (*b* = 0.3485, SE = 0.0560, *t* = 6.23, *p* < 0.001), Reward (*b* = −0.3723, SE = 0.0603, *t* = −6.17, *p* < 0.001), and meaningful work (*b* = 0.1506, SE = 0.0457, *t* = 3.29, *p* = 0.001) retained statistically significant associations. Thus, the substantive pattern did not depend solely on combining Effort and Reward into a single ratio score.

These findings supported Hypotheses 1–4.

### 4.5 Testing the moderating effect of social support

PROCESS Model 14 was used to estimate the conditional indirect association. As shown in Table 5, the interaction between meaningful work and social support was statistically associated with emotional exhaustion (*b* = −0.0982, SE = 0.0330, *t* = −2.9760, *p* = 0.0031), indicating that the strength of the meaningful work–emotional exhaustion association differed across levels of social support.

**TABLE 5:** Moderated mediation analysis results.

Path	*b*	SE	t	*P*	LLCI	ULCI
Meaningful work × Social support → Emotional exhaustion	−0.0982	0.0330	−2.9760	0.0031	−0.1631	−0.0334
**Social support**	**Effect**	**SE**	**t**	**p**	**LLCI**	**ULCI**
Conditional effects of meaningful work on emotional exhaustion						
Low (−1 SD)	0.3293	0.0485	6.7957	<0.001	0.2341	0.4245
Mean	0.2287	0.0394	5.8017	<0.001	0.1513	0.3062
High (+1 SD)	0.1282	0.0552	2.3246	0.0205	0.0198	0.2366
**Social support**	**Effect**	**BootSE**	**BootLLCI**	**BootULCI**		
Conditional indirect effects						
Low (−1 SD)	0.1919	0.0352	0.1265	0.2645		
Mean	0.1333	0.0275	0.0821	0.1896		
High (+1 SD)	0.0747	0.0331	0.0105	0.1405		
**Index**	**BootSE**	**BootLLCI**	**BootULCI**			
Index of moderated mediation						
−0.0572	0.0197	−0.0982	−0.0212			

Simple slope analyses further showed that the positive relationship between meaningful work and emotional exhaustion was stronger under low levels of social support (*b* = 0.3293, *p* < 0.001) than under high levels of social support (*b* = 0.1282, *p* = 0.0205). Figure 2 illustrates the moderating effect of social support.

**FIGURE 2:**
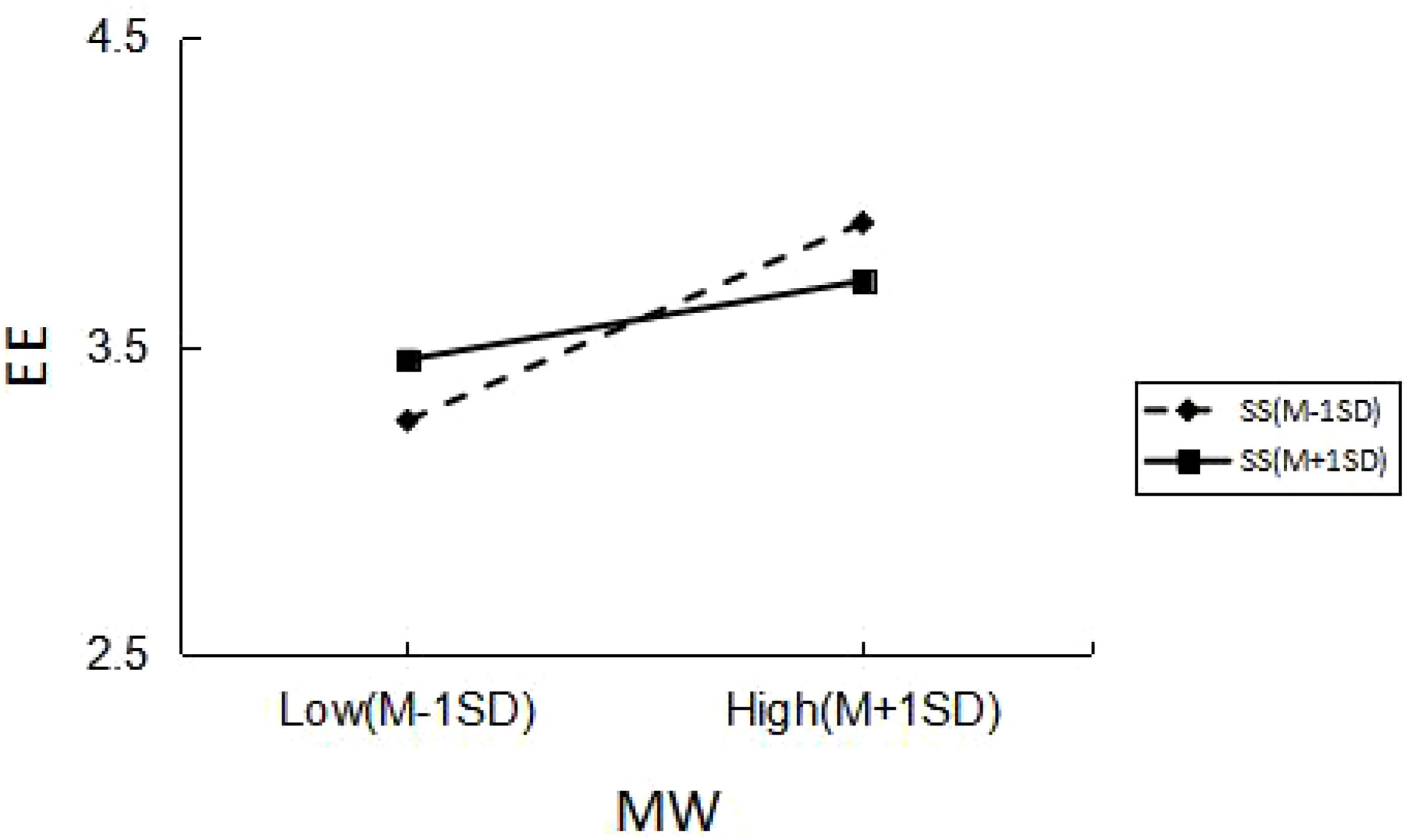
Moderating effect of social support on the relationship between meaningful work and emotional exhaustion. MW, meaningful work; EE, emotional exhaustion; SS, social support.

The conditional indirect-association estimates became smaller as social support increased. The estimate was largest at low social support (estimate = 0.1919, 95% CI [0.1265, 0.2645]) and smallest at high social support (estimate = 0.0747, 95% CI [0.0105, 0.1405]).

Moreover, the index of moderated mediation was statistically different from zero (index = −0.0572, BootSE = 0.0197, 95% CI [−0.0982, −0.0212]). In plain language, the negative index indicates that each one-unit increase in reported social support was associated with a 0.0572-unit decrease in the model-estimated indirect association between ERI and emotional exhaustion through meaningful work. Thus, the indirect association was strongest at low social support and weakest at high social support. Because the bootstrap interval excluded zero, this rate of change was statistically distinguishable from zero within the specified model; it does not demonstrate that increasing support causally reduces exhaustion.

Therefore, Hypothesis 5 was supported.

These findings indicate that the positive association between meaningful work and emotional exhaustion was weaker at higher reported levels of social support. This conditional pattern does not establish that support causally reduced exhaustion; reverse or reciprocal relationships remain possible.

## 5 Discussion

The moderated mediation analysis further showed a negative index: as reported social support increased, the model-estimated indirect association between effort–reward imbalance and emotional exhaustion through meaningful work became progressively smaller. This pattern indicates attenuation of the conditional indirect association at higher levels of support, but it does not establish a causal buffering effect.

### 5.1 Role of meaningful work in chronically demanding work contexts

The present study found that effort–reward imbalance was positively associated with emotional exhaustion among border-region teachers. This pattern is consistent with ERI theory and previous research showing an association between prolonged imbalance in occupational effort and reward and greater psychological strain (Siegrist, 1996; van Vegchel et al., 2005). Because the data were cross-sectional, the finding should not be interpreted as evidence that ERI temporally preceded or caused emotional exhaustion.

More importantly, meaningful work accounted for a significant indirect statistical association between ERI and emotional exhaustion. This pattern differs from the assumption that meaningful work necessarily functions as a uniformly protective psychological resource. Existing studies have generally emphasized positive associations of meaningful work with motivation, engagement, and well-being (Rosso et al., 2010; Steger et al., 2012; Lysova et al., 2019). The current findings suggest that the psychological correlates of meaningful work may vary across occupational contexts, particularly in environments characterized by persistent stress and limited recovery opportunities. However, alternative temporal orderings and reciprocal relationships remain possible.

One possible, non-causal interpretation is that teachers who strongly value the significance of their work may also report sustained emotional investment when occupational conditions remain unfavorable. In this context, meaningful work may coexist with professional commitment and greater emotional burden. This interpretation does not imply that meaningful work is harmful or that it increases exhaustion; rather, the observed positive association may reflect the way meaning and emotional investment coexist under chronic occupational strain.

This interpretation may be particularly relevant in educational environments where teachers face continuous organizational demands while possessing relatively limited institutional resources. Previous research has shown that teachers’ emotional experiences are closely connected to long-term professional commitment and moral responsibility within school settings (Hargreaves, 1998; Kelchtermans, 2005). Under such conditions, maintaining a strong sense of work meaning may encourage teachers to remain emotionally engaged despite ongoing stress exposure. Over time, however, sustained emotional involvement without adequate recovery may gradually contribute to emotional depletion.

### 5.2 The conditional role of social support

The association between meaningful work and emotional exhaustion varied significantly across levels of social support and was weaker at higher levels of support. This pattern is consistent with, but does not by itself demonstrate, a buffering interpretation. Supportive interpersonal and organizational environments may be one contextual factor associated with lower emotional costs of sustained work involvement.

This result is generally consistent with previous research on the stress-buffering hypothesis (Cohen and Wills, 1985; Bakker and Demerouti, 2007), but the present cross-sectional data establish only that the association differed across reported levels of support. Teachers experiencing less emotional exhaustion may also be better able to perceive, seek, or mobilize support from colleagues, administrators, and social networks. Longitudinal evidence is needed to distinguish these temporal possibilities.

Importantly, the present findings suggest that social support may not simply reduce stress directly. Rather, supportive environments may help teachers maintain professional involvement in a more sustainable manner. Even when teachers strongly value the meaning of their work, adequate interpersonal and organizational support may reduce excessive emotional burden by providing emotional reassurance, practical assistance, and opportunities for psychological adjustment. In this sense, social support may function as an important contextual condition influencing how teachers experience and manage sustained occupational demands.

The border-region location should therefore be understood primarily as a sampling characteristic. Because no non-border comparison group and no direct measures of geographic dispersion, multiethnic composition, or cross-cultural contact were included, the observed interaction cannot be attributed to those contextual features.

### 5.3 Theoretical implications

The present study contributes to the literature in several ways. First, the findings extend existing research on meaningful work by suggesting that its psychological associations may vary across occupational contexts. Previous studies have primarily emphasized positive associations of meaningful work with motivation, engagement, and well-being (Steger et al., 2012; Allan et al., 2019). In the present sample, meaningful work was also associated with greater emotional burden under conditions characterized by persistent occupational strain and insufficient recovery opportunities. This correlational pattern highlights the importance of considering contextual and organizational conditions when examining the psychological correlates of meaningful work.

Second, the present study extends ERI research within an educational sample. The observed indirect association is consistent with the possibility that teachers’ interpretations of work meaning are linked to how occupational imbalance and emotional burden coexist. It should not be interpreted as evidence that meaningful work is a causal mechanism through which ERI produces exhaustion.

Third, the study contributes to research on teacher well-being by emphasizing the relevance of social and organizational support systems. The observed interaction indicates that the association between meaningful work and emotional exhaustion was weaker at higher levels of social support. This pattern is consistent with stress-buffering perspectives (Cohen and Wills, 1985), but the cross-sectional data do not demonstrate that social support caused a reduction in emotional burden.

Finally, the findings underscore the value of examining teacher well-being within broader institutional and contextual environments. The present border-region sample offers one setting in which individual psychological characteristics and organizational conditions can be considered together; the data do not establish that this setting differs systematically from non-border educational contexts.

### 5.4 Practical implications

The present findings also provide several practical implications for educational administrators and school organizations. First, reducing teachers’ emotional exhaustion may require more than simply lowering workload or short-term occupational stress. The current findings suggest that teachers who strongly value the meaning of their work may continue maintaining high levels of emotional involvement even under stressful working conditions. Therefore, efforts to improve teacher well-being should not focus solely on enhancing motivation or professional commitment, but should also consider whether teachers have sufficient opportunities for emotional recovery and psychological adjustment.

Second, the findings highlight the importance of supportive school environments. In educational settings characterized by limited institutional resources, interpersonal and organizational support may become particularly important for maintaining teachers’ psychological well-being. Schools may therefore benefit from strengthening collegial communication, supportive leadership practices, and peer-support systems that help teachers cope with long-term emotional demands. Providing opportunities for emotional expression, collaborative interaction, and professional support may help reduce the accumulation of emotional exhaustion over time.

Third, the observed interaction shows that teachers reporting higher social support also exhibited a weaker positive association between meaningful work and emotional exhaustion. Educational management may therefore consider the relational climate within schools while recognizing that the present cross-sectional results do not establish that supportive environments causally improve teachers’ psychological adjustment.

Finally, the findings may inform future educational policy research in border-region settings. Because this study did not include a non-border comparison group, recommendations should focus on the measured work conditions–such as effort–reward imbalance and available support–rather than attributing the observed associations to border-region status itself.

### 5.5 Limitations and future directions

Several limitations of the present study should be acknowledged. First, the cross-sectional design precludes conclusions about temporal ordering or causation. Although the indirect and conditional associations were theory-guided, teachers who experience greater meaning may evaluate effort and reward differently, and teachers experiencing less exhaustion may be better able to perceive or mobilize social support. Meaningful work could also plausibly moderate, rather than mediate, the ERI–exhaustion association. Longitudinal cross-lagged, experimental, or intensive repeated-measure designs are needed to compare mediation, moderation, reverse-direction, and reciprocal models. In addition, although distributional and Mahalanobis-distance diagnostics were used during screening, maximum-likelihood and regression-based estimates may remain sensitive to substantial distributional departures; bootstrap confidence intervals only partially reduce reliance on normal-theory inference.

Second, all variables were measured concurrently using self-report questionnaires, creating a risk of common method variance. Harman’s single-factor test indicated that the first unrotated component explained 30.110% of the total variance, suggesting that a single general component did not dominate the item covariance. However, this coarse diagnostic cannot exclude common method bias, and the observed direct, indirect, and conditional associations may still be inflated. Future studies should combine temporally separated measurements, multiple data sources, and marker-variable or latent-method-factor approaches.

Third, participants were recruited from border-region schools, but the study included neither a non-border comparison group nor direct measures of geographic dispersion, ethnic composition, or cross-cultural contact. Border-region status should therefore be interpreted as a sampling characteristic rather than a demonstrated explanatory context. The findings may not generalize to other educational or occupational settings, and comparative multisite studies are needed to test whether the same associations occur in border and non-border schools.

Finally, the present findings suggest several directions for future research on meaningful work. Existing studies have primarily emphasized its positive psychological functions. However, the associations observed in the current cross-sectional study may vary across occupational conditions and recovery opportunities. Future longitudinal research should examine how supportive organizational environments, recovery experiences, and long-term occupational stress are associated with meaningful work and emotional exhaustion over time.

## Data Availability

The data that support the findings of this study are available from the corresponding author upon reasonable request.
